# Design of Signal Generators Using Active Elements Developed in I3T25 CMOS Technology Single IC Package for Illuminance to Frequency Conversion

**DOI:** 10.3390/s20041198

**Published:** 2020-02-21

**Authors:** Roman Sotner, Jan Jerabek, Ladislav Polak, Vilem Kledrowetz, Roman Prokop

**Affiliations:** 1 Department of Radio Electronics, Faculty of Electrical Engineering and Communication, Brno University of Technology, Technicka 3082/12, 61600 Brno, Czech Republic; polakl@feec.vutbr.cz; 2 Department of Telecommunications, Faculty of Electrical Engineering and Communication, Brno University of Technology, Technicka 3082/12, 61600 Brno, Czech Republic; jerabekj@feec.vutbr.cz; 3 Department of Microelectronics, Faculty of Electrical Engineering and Communication, Brno University of Technology, Technicka 3058/10, 61600 Brno, Czech Republic; kledrowetz@feec.vutbr.cz (V.K.); prokopr@vutbr.cz (R.P.)

**Keywords:** CMOS active element, comparator, electronic adjusting, frequency tunability, functional generator, illuminance sensing, integrator, square and triangular waves

## Abstract

This paper presents a compact and simple design of adjustable triangular and square wave functional generators employing fundamental cells fabricated on a single integrated circuit (IC) package. Two solutions have electronically tunable repeating frequency. The linear adjustability of repeating frequency was verified in the range between 17 and 264 kHz. The main benefits of the proposed generator are the follows: A simple adjustment of the repeating frequency by DC bias current, Schmitt trigger (threshold voltages) setting by DC driving voltage, and output levels in hundreds of mV when the complementary metal-oxide semiconductor (CMOS) process with limited supply voltage levels is used. These generators are suitable to provide a simple conversion of illuminance to frequency of oscillation that can be employed for illuminance measurement and sensing in the agriculture applications. Experimental measurements proved that the proposed concept is usable for sensing of illuminance in the range from 1 up to 500 lx. The change of illuminance within this range causes driving of bias current between 21 and 52 μA that adjusts repeating frequency between 70 and 154 kHz with an error up to 10% between the expected and real cases.

## 1. Introduction

The triangular and square wave waveform generators are key subparts of many analog and mixed circuits (clock generation, pulse width modulation, DC-DC converters, etc.) [1]. However, not all solutions are suitable for electronically controllable applications. Many standard and modern active elements (AEs) [2,3] fulfill requirements for their implementations in these generators. However, using them leads to very limited electronic adjustability of the repeating frequency in the generators (e.g., see comparison in [4]). Such phenomenon is caused by the implementation of nonadjustable active elements in key parts of a generator (e.g., see once again comparison in [4], where the AD844 device is used in an integrator or comparator).

We compared the features of solutions presented in this area recently (see Table 1). It is very hard to estimate the occupied area of silicon or printed circuit board (PCB) of some solutions [5,6,7,8,9,10,11,12,13,14,15,16,17,18,19,20,21,22,23,24,25,26] because many circuits are simulated without design of die layout or PCB. Therefore, we can evaluate only the complexity of real circuitry based on available information.

Many concepts use standard commercially available AEs (e.g., operational transconductance amplifiers (OTAs) [5,6], current conveyors [10], operational amplifiers [14,25], variable gain amplifiers [14,16,17], or their easily accessible models, i.e., internal topologies). In numerous works, the active subparts of basic building structures [2,3] are understood as a single element [7,8,9,11,12,13,15,18,19,20,21,22,23]. It substantially changes view on the circuit and credibility of results when these concepts are presented as: (a) Simulated models only, (b) real so-called “behavioral models” [2,3] for experiments (based on off-the-shelf components), and (c) real fabricated complementary metal-oxide semiconductor (CMOS) cells encapsulated in a single integrated circuit (IC) package. However, many AEs are used and designed on hypothetical level [7,8,9,11,12,13,15,18,19,20,21,22,26] without fabrication in the form of IC. In fact, many of these devices include several functional active subparts. In some cases, the experimental verification was obtained from the behavioral model of a hypothetical AE [9,13,16,17,18,19,22,23,26]. Generally, this method is useful and frequently used by researchers. Unfortunately, the obtained experimental results follow the trends from simulation of hypothetical CMOS internal structures rarely due to unsuitability of the off-the-shelf commercial AEs for the supposed operational bands (e.g., see [21]). Next, for different conditions, many recent works present more simulation-based results than measurement ones (high frequencies are unavailable in real cases due to high parasitic performances), see for example [21,22]. Numerous experimental solutions [13,18,19,21] have really extensive circuitry because of implementation of commercially available AEs in behavioral model of the hypothetical AE. Based on the analysis of solutions included in Table 1, only one solution includes fabricated AEs in the frame of CMOS technology in the form of so-called modular approach (several types of AEs with suitable and/or variable interconnection of their terminals [27,28,29,30]).

The main goal of our work is to realize tunable generators (available simultaneously) with the following features:

(a) A simple electronic control of the repeating frequency (*f*_0_) by bias current,
(b) Bias voltage driving threshold voltages of the Schmitt trigger,
(c) Hypothetical elements are not used,
(d) Simple and compact solutions (a single package employed) minimized by a fabricated IC device (there are not similar designs except of [10], but in [10] too many passive elements are required),
(e) High amplitude of the output voltage levels in comparison with other solutions, where output levels are in tens of mV (similarly to as in simulated waveforms, e.g., see [21]).

Our solutions bring more compact real circuitry of triangular and square wave generators preserving features of already known concepts, but significantly reduce the number of required active components (comparable to the rest of solutions, see Table 1) and area of the circuitry. Concepts presented in [5,6,14,16,17,24,25] use standard active elements that are commercially available (easily accessible for designers-significant advantage). However, there are some disadvantages, namely: High power consumption (information is not available in many papers), high area requirements, high number of passive elements for serial production of a final application. Solutions presented in [7,8,9,11,12,13,15,18,19,20,21,22,23,26] have core in hypothetical (not fabricated as a single chip) subparts creating a complex active element. Many of these solutions are constructed as behavioral models using commercially available parts and tested experimentally. Quite high power consumption and high supply voltage are typical issues of these approaches but the intention of such designs is only a verification of theoretical presumptions. Only a limited number of solutions really fabricated as IC and using external passive elements has been found in recent literature. However, this is the best way to minimize power consumption and achieve good performance in variability (having external capacitor and resistors of comparator for simple modification of operational bandwidth) as our findings indicate. Unfortunately, solution [10] (using also a single IC package as our proposal) requires too many external passive elements (8 in [10], three in our cases).

From a practical point of view (number of real IC packages in measurements), our solution seems to be the simplest. Moreover, there is a limited number of electronically tunable generators employing integrator with the current conveyor [10,14,15,17,24,25,26] in standard [10] or controllable form (gain [2], [3] adjusted in [14,15,17,25]. These circuits utilize linear operation of the current conveyor. However, operation out of its linear range, which is helpful to obtain high output signal levels (tens of mV), in current conveyor-based integrator (see Table 1) was not studied in detail so far, except in [10] and [16]. The saturated integrator with conveyor allows similar derivation of design equation as in the case of OTA, where the current (charging *C*) is directly proportional to bias current [5,6,7,8,9,11,13,18,23]. There is a very limited number of integrated generators as XR8038, XR2206, etc. However, many of them are several years obsolete and unavailable (MAX038). Compared to the proposed circuits, their features are very similar (e.g., operational frequencies) but power consumption is several times higher (hundreds of mW) in these bipolar technologies.

The remaining parts of this paper are organized as follows. Principle of the used AE in the frame of a single IC package is introduced in Section 2. Section 3 forms the main core of the paper and brings two circuitries of triangular and square wave generator including their experimental verification. Differences between the proposed concept and the most similar solutions, as well as general differences between the used tunability principle and standard approaches are discussed in Section 4. A very simple application of the generator for sensing purposes (converting illuminance into the repeating frequency) is shown in Section 5. This paper is concluded in Section 6.

## 2. Description of Active Elements

**Figure 1:**
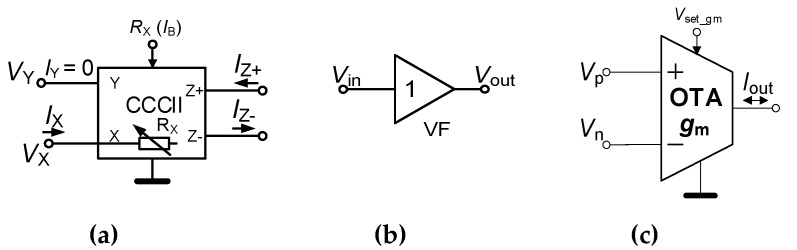
Active elements (AEs) in a single integrated circuit (IC) package (device) developed in I3T 0.35 μm complementary metal-oxide semiconductor (CMOS) process: (**a**) current controlled current conveyor of second generation (CCCII); (**b**) voltage follower (VF) and (**c**) operational transconductance amplifier (OTA).

Our design utilizes three active subparts of an IC fabricated in I3T25 0.35 μm (ON semiconductor) CMOS technology. The active cells, included in a single IC package, are shown in Figure 1. The current controlled current conveyor of second generation (CCCII) [2,3] is shown in Figure 1a. Its inter-terminal relations are typical and useful for many applications: *I*_Z+_ = *I*_X_, *I*_Z−_ = −*I*_X_ (note that the unused output Z is grounded), *V*_X_ = *V*_Y_ + *R*_X_∙*I*_X_ denotes a case when current flowing from the X terminal, otherwise *V*_X_ = *V*_Y_ (X floating). The resistance *R*_X_ can be electronically controlled by the bias current *I*_B_ as: *R*_X_ ≅ 3.5 × *I*_B_^−1/2^. More details and the CMOS topology of CCCII can be found in [4]. The voltage follower (VF) with transfer function *V*_out_ = *V*_in_ has been also included in the same IC (once again, more details are in [4]). The OTA represents the last important active cell of the IC. Thanks to their features, based on the multiplying core, it offers unique but simple change of its output current polarity (*I*_out_ = ± (*V*_p_ − *V*_n_)∙*g*_m_), where *g*_m_ ≅ 1.8 × 10^−3^∙*V*_set_gm_. Further details and CMOS topology are presented in [27,30]. According to the theory, CCCII and OTA cells can be easily used for construction of the required building blocks of the generator [1,5].

## 3. Generator Based on Schmitt Trigger with OTA and Current Controlled Integrator Using CCCII

**Figure 2:**
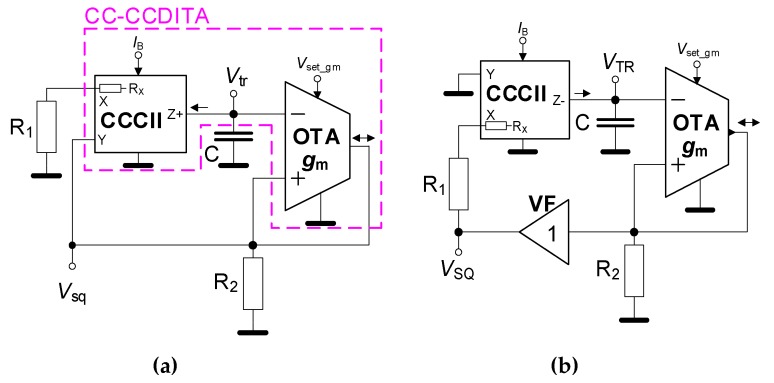
The proposed topology of the current-controlled generator with: (**a**) An integrator having input terminal in Y of CCCII and (**b**) an integrator having input terminal in X of CCCII through resistor *R*_1_.

There are two possible solutions that can be found by suitable interconnection of OTA and CCCII parts as shown in Figure 2. The OTA and *R*_2_ create a very simple Schmitt trigger [1]. Note that many researchers refer to the interconnection of the CCCII and OTA as current conveyor transconductance amplifier (CCTA) family of AEs [2,3]. In our particular case, it is a current controlled current conveyor differential input transconductance amplifier (CC-CCDITA) as indicated in Figure 2a by the purple color.

The basic idea of operation leading to design equations is almost identical for both solutions (especially in the case of Schmitt trigger). The input thresholds and output voltages of the Schmitt trigger are in relation:

(1) $$\pm V_{tr}=\mp V_{sq}\left[\frac{g_{m}R_{2}-1}{g_{m}R_{2}}\right],$$

that means controllability of threshold voltages by *g*_m_ (the DC driving voltage is denoted by *V*_set_gm_). The maximal change of the voltage across the capacitor is expressed as:

(2) $$\Delta v_{C}=v_{C}(t=T_{0}/2)-v_{C}(t=0)=\frac{I_{C\max}}{C}\frac{T_{0}}{2}.$$

The complete determination of Equation (2) was shown in [14]. However, both specific solutions in Figure 2 have a significant nonstandard difference from the concept presented in [14]. Due to the large level of OTA’s output current flowing through the resistor *R*_2_, the maximal voltage *V*_sq_ reaches the saturation level slightly below the approximated ±1.5 V at the supply voltage of ±1.65 V. Therefore, when *V*_Y_ > ±0.5 V (for CCCII; in fact, when *V*_sq_ > ±0.5 V as obvious) then the maximal value of the current charging the *C* is given as: *I*_Cmax_ = 10∙*I*_B_ (exactly valid for *I*_B_ ≤ 120 μA). It is not typical in the case of similar solutions (integrator using current conveyor) because *I*_Cmax_ is frequently given by the saturation voltage (*V*_sq_) and it is, therefore, allowed only in linear dynamics of the current conveyor (see [14,15,17]). The constant value of 10 results from mirroring gains of the internal topology of the CCCII cell [4]. Hence, *R*_1_ is not considered in the equations and it allows generating larger output levels than in the case working within linear dynamics of the AE. In our nonlinear case, charging and discharging the current level for *C* is the most important in comparison to linear applications. Note that the following considerations are valid for duty cycle 50%. In general, the voltage across capacitor is:

(3) $$\Delta v_{C}=2V_{tr}=\frac{I_{C\max}}{C}\frac{T_{0}}{2}.$$

Rearrangement of Equation (3) and corresponding equations yield the final form of repeating frequency (*f*_0_) as:

(4a) $$f_{0}=\frac{I_{C\max}}{4C\cdot V_{tr}}≅\frac{10\cdot I_{B}}{4C\cdot V_{tr}},$$

(4b) $$f_{0}≅\frac{10\cdot I_{B}}{4C\cdot V_{SQ}}\left(\frac{g_{m}R_{2}}{g_{m}R_{2}-1}\right).$$

Term *f*_0_ = 10∙*I*_B_/(4∙*C*∙*V*_tr_) in Equation (4a) represents the simplified way to determine *f*_0_, where the value of *V*_tr_ level must be known. Equation (4b) supposes a constant saturation level of *V*_sq_. The second version of the generator shown in Figure 2b has almost identical description as the concept presented in Figure 2a. We suppose the validity of Equations (1)–(3) and expectation of saturating *V*_sq_ (*V*_Y,X_ > ±0.5 V). Hence, the equation for *f*_0_ has an identical form as Equation (4). The difference is in the inverted polarity of CCCII output Z and additional voltage follower in the circuitry.

**Figure 3:**
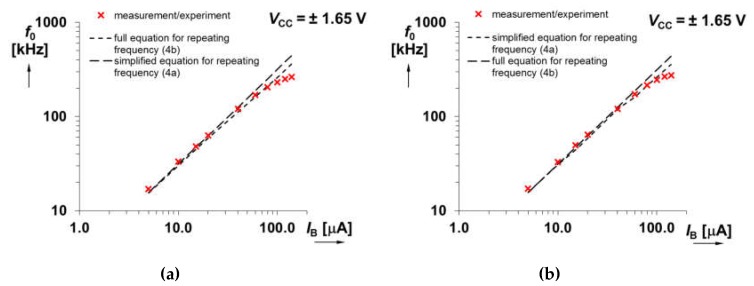
Comparison of calculated and measured dependence of *f*_0_ on current *I*_B_ for solution with: (**a**) Integrator having input terminal in Y of CCCII (Figure 2a), (**b**) integrator having input terminal in X of CCCII through resistor (Figure 2b).

**Figure 4:**
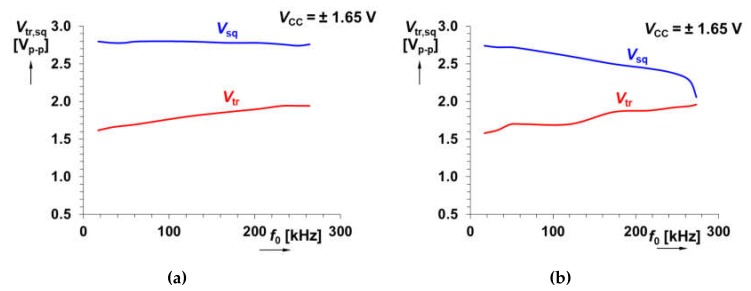
Measured dependence of output levels on *f*_0_ for solution shown in: (**a**) Figure 2a, (**b**) Figure 2b.

Our adjustment of *f*_0_ targets on the frequency bandwidth is between 20 and 200 kHz. We suppose *g*_m_ ≅ 500 mS (*V*_set_gm_ = 0.25 V) [27,30], next *R*_1_ = 560 Ω and *R*_2_ = 4.7 kΩ. Therefore, this operational frequency range is theoretically available for *C* = 1 nF and *I*_B_ from 6 up to 64 μA. We tested the range of *I*_B_ from 5 up to 140 μA. It allows theoretical readjustment of *f*_0_ (calculated with the full form of Equation (4) from 16 up to 440 kHz. The experimental tests yield results from 17 up to 264 kHz. Dependence of *f*_0_ on *I*_B_ for both solutions (see Figure 2) is given in Figure 3. Differences between the curves obtained for the target range of adjustability is negligible. The reason for small deviations of measured points from linear trace for higher values of *I*_B_ is limited validity of *I*_Cmax_ = 10∙*I*_B_. At *I*_B_ higher than 120 μA, the output current (z terminal) of the CCCII is depending on *I*_B_ nonlinearly. On the other hand, the difference can be seen for the curves of output levels of square and triangular wave versus *f*_0_ (Figure 4).

The first circuit has more stable output levels (*V*_sq_ = 2.74 → 2.8 V_p-p_), *V*_tr_ = 1.62 → 1.94 V_p-p_) in the observed bandwidth than the second one (*V*_sq_ = 2.06 → 2.74 V_p-p_), *V*_tr_ = 1.58 → 1.96 V_p-p_). Variations of voltage levels (especially triangular wave) are typical for designs operating at the border of high frequency limits of AEs for large signals (units of V) with a wideband spectral characteristic [12,17].

The operating temperature influences mainly the input resistance of the current conveyor CCCII (X—terminal has small-signal resistance *R*_X_) and the transconductance *g*_m_ of the OTA part. Fortunately, the *R*_X_ is not connected with the definition of *f*_0_, see Equations (4) and (5), because it is not present in the integrator transfer function due to special operation of the integrator (charging C to maximal current *I*_Cmax_ = 10∙*I*_B_). The *g*_m_ of the CMOS OTA cell should be considered carefully. Datasheet of the fabricated device, based on corner simulations, indicates *g*_m_ variation ±10% for temperature change 20–40 °C. Considering these dispersions in Equation (4), the lowest frequency for *I*_B_ = 5 μA varies between 14 and 17 kHz and the highest frequency for *I*_B_ = 100 μA, where dependency is still almost linear (see Figure 3), should be estimated between 291 and 339 kHz. Experimentally obtained results at room temperature follow this behavior.

**Figure 5:**
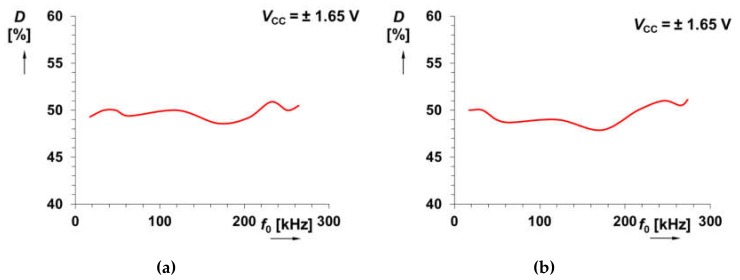
Results from measurements showing the dependence of duty cycle on the change of repeating frequency *f*_0_ for the solution presented: (**a**) in Figure 2a, (**b**) in Figure 2b.

**Figure 6:**
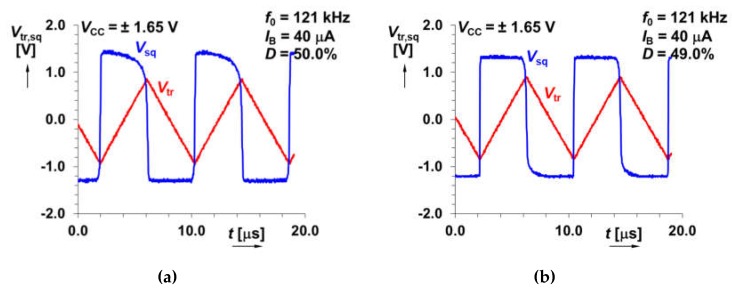
An example of the measured output responses for solution presented: (**a**) in Figure 2a, (**b**) Figure 2b.

Figure 5 depicts the symmetry of the output waveforms. The duty cycle value fluctuates around 50% with maximal deviation ±2%. The duty cycle control can be easily provided by an additional DC current source (see in [19] and [22]). The examples of output waveforms for *I*_B_ = 40 μA (*f*_0_ = 121 kHz) are given in Figure 6. The power consumption of the circuits in Figure 2a,b, at supply voltage ±1.65 V, reaches maximally 67 and 86 mW, respectively. Our IC design occupies 0.28 and 0.37 mm^2^.

## 4. Comparison of the Proposed Concepts with Previous Works

The comparison of the proposed solution in Figure 2a and circuit in [25] shows topological similarity. However, the output level of triangular wave in [25] is decreased by a resistive divider (not used in our case). Moreover, in the final circuit design, standard commercially available discrete devices (CCII AD844 and OTA LM13700) are utilized. The tunable version of the topology in [25] supposes the impact of *R*_X_ (ideally as much linear as possible and without any further limitation of charging current) on *f*_0_. Our solutions use a different way of control (saturated output current defined by the DC bias adjustment, see the sixth column in Table 1). This leads to significantly increased level (more than 1.5 V_p-p_ over the tunable range of frequency) of the output waveforms (especially triangular wave) in comparison to the previously published CMOS designs (typically less than 1 V_p-p_, for example [11,12,22,23], or even in tens of mV in [21]). The circuit in Figure 2b employs the opposite direction of current charging the capacitor.

Hence, the topology also uses the current conveyor cell having negative polarity of z terminal, as well as an additional voltage buffer in loop. Our solutions include intentionally overexcited integrator with input voltage levels that generates maximal charging current (for capacitor) equal to saturation levels determined by the bias current setting. That is the main difference between our solution and that one [25], where the charging current is determined by the value of linear *R*_X_ [25].

The active element in [25] itself is not limiting current levels, similarly as in [14,15,17,24]. Analysis of our both solutions, presented in this paper, yields different equations for *f*_0_ (including different parameters for adjustment of threshold voltages of the comparator) than analysis presented in [25].

## 5. Illuminance to Frequency Converter—A Sensing Application

**Figure 7:**
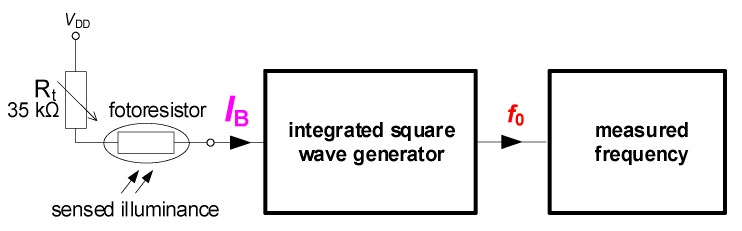
The proposed illuminance to frequency converter for sensing applications.

In this section, we present a concept employing the concurrent-controlled generator presented above to convert illuminance to frequency. Such a converter, which the block diagram is shown in Figure 7, is useful for simple low-cost sensing applications. The read-out system is divided into two main parts: Transformation of illuminance (*IL*) to bias current (*I*_B_) required for driving of the generator and generator producing waveforms with corresponding repeating frequency *f*_0_. Thanks to this concept, fluctuances of output amplitudes are not important because information about illuminance is represented as *f*_0_.

**Figure 8:**
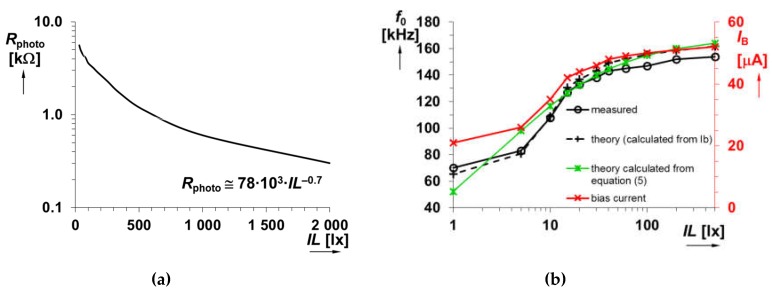
Analysis of sensing application: (**a**) Dependence of photoresistor resistivity on illuminance and (**b**) dependence of bias current and repeating frequency of the generator (see Figure 2b) on illuminance.

The driving of *I*_B_ by variable resistance of a photoresistor (*R*_photo_) is expressed by Equation *I*_B_ = (*V*_DD_ − *V*_GS_)/(*R*_t_ + *R*_photo_), where *V*_DD_ is the supply voltage (asymmetric: 3.3 V, i.e., 2 × 1.65 V) and *V*_GS_ is the gate-source voltage of internal CCCII bias current diode reference (a part of the current mirror). The value of *V*_GS_ also depends on transconductance, threshold voltage, and also on the aspect ratio of transistor (in our case 1.4 V was obtained for *I*_B_ = 50 μA). In our work, the photoresistor type of LDR5516 [31] is utilized. The measured dependence of the resistivity of photoresistor on illuminance is shown in Figure 8a. As it can be seen, the obtained curve is highly nonlinear (our measurements show approximate relation equal to *R*_photo_ ≅ 78 × 10^3^∙*IL*^−0.7^) and it was tested for the range of *IL* from 30 to 2000 lx (*R*_photo_ 6 kΩ → 300 Ω).

It is important to note that there is no further information provided by the producer [32]. A set of experimental measurements was performed to determine the relation between *f*_0_ and *IL* that clearly indicates dependence of *f*_0_ on *IL* (valid for light in the visible part of spectrum [33]) as visible from Equation (5):

(5) $$f_{0}≅\frac{10\cdot \left(\frac{V_{DD}-1.4}{R_{t}+R_{photo}}\right)}{4C\cdot V_{SQ}}\left(\frac{g_{m}R_{2}}{g_{m}R_{2}-1}\right)≅\frac{10\cdot \left(\frac{V_{DD}-1.4}{R_{t}+78\cdot 10^{3}\cdot IL^{-0.7}}\right)}{4C\cdot V_{SQ}}\left(\frac{g_{m}R_{2}}{g_{m}R_{2}-1}\right).$$

The resistor *R*_t_ had a value of 35 kΩ in order to set appropriate conditions for operation in tens of μA of the bias current. The *R*_photo_ varied its value from 50 to 1.5 kΩ when it was exposed by *IL* in the range from 1 to 500 lx. Compared to the whole range presented in Figure 8a, it is significantly a narrower range. Nevertheless, it is sufficiently representing standard daylight room conditions. These values of resistance control *I*_B_ between 21 and 52 μA results into an experimentally gained tunability range of *f*_0_ from 70 up to 154 kHz. A simple theoretical estimation based on knowledge of upper and lower values of *I*_B_ predetermines the value of ideal *f*_0_ into the range from 63 up to 157 kHz. Using Equation (5), we obtained values between 52 and 164 kHz. In Figure 7, the “integrated square wave generator” block denotes the circuit from Figure 2b.

**Figure 9:**
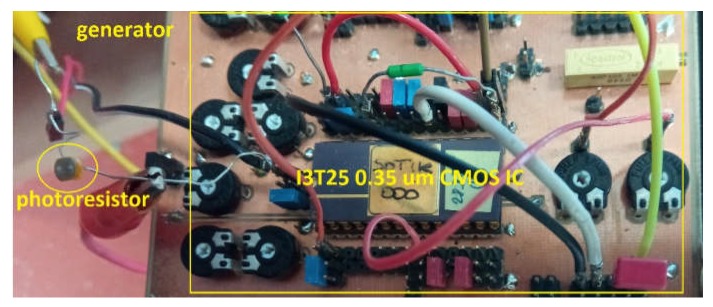
Photography of tested prototype of illuminance sensor converting resistance value to repeating frequency.

Its design and setting are the same as was presented above. Figure 9 depicts the PCB realized for verification purposes of the proposed illuminance to frequency converter suitable for sensing applications.

The error between the measured and ideal values does not overcome ±5% when considering *IL* having a value above 5 lx and ±7–10% for the range below this value (see Figure 8b). Moreover, it is visible that the error between experimental and theoretical curves, calculated from Equation (5), achieves very similar values (around ±5%) but results for values of *IL* below 5 lx are obviously influenced by larger deviations (more than 15%).

### Sensing Applications—A Brief Discussion of the State-of-the-Art

The area of simple electronics sensing read-outs focuses on the sensing of physical quantities [34], such as temperature [35], mechanical pressure [36], acoustic pressure [37], electromagnetic field [38], humidity [39], gas [40], bio-signal [41], or capacity [42,43], etc. However, the detection and measurement of light and illuminance [44] received quite limited attention in recent works. There are only works targeting on exposed area analysis [45,46] and technological (material) design of photosensors [47]. A quite complex device for illuminance measurement was introduced in [48].

However, such concept requires microcontroller with a special development board and additional software detection algorithm for the signal processing. Of course, it can improve the accuracy and sensitivity, but results in high costs and complexity for sensing applications utilized for simple purposes. Therefore, our presented read-out system can be useful for low-cost designs. The proposed converter can be applied in simple or complex systems for illuminance measurement and sensing in the agriculture applications, as shown for example, in [49].

Further extension of our work can be targeted into the fields of biological and material sciences, where analysis of concepts using light sources and detectors is very important. There are micro-optical sensors useful for biological applications (analysis of bio tissues by light of various wavelengths) [50,51]. However, these sensors represent specific single-purpose and expensive devices.

## 6. Conclusions

In this paper, a design of triangular and square wave generators with low number of packages (a single IC) was presented. The bias current (*I*_B_) adjusted from 5 up to 140 μA has been used for tunability of repeating frequency (*f*_0_) of the generators. Compared to standard solutions built with an integrator based on current conveyors, we expect a saturated AE of the integrator that simplifies the design. The maximal value of the charging current (*I*_Cmax_) is defined by the maximal output current (*I*_Z±_) of the CCCII directly relating with bias driving (*I*_Cmax_ ≅ 10∙*I*_B_), which brings larger output levels than in standard cases. The operational bandwidth of *f*_0_ tunability was found between 17 and 264 kHz. The proposed solutions allow achieving a very stable duty cycle with only 2% error in the observed range. The power consumption reaches values from 58 up to 90 mW due to high output voltage levels (as shown in [19]). Note that many known topologies of generators (for example [24,26,32]) can be modified in order to provide simple electronic tuning but specified types of the used AEs do not allow it. Experimental results confirmed operationability of our circuits with a compact size, i.e., occupying only a single IC package, for simple designs. All conclusions presented in this paper are supported by experimental measurements of a circuit with a single IC device proposed and fabricated by us in a I3T25 0.35 μm ON semiconductor CMOS process.

In addition, in this paper, an illuminance to frequency converter utilizing one of our proposed generators (see Figure 2b) was introduced for simple sensing application purposes. The measured illuminance between 1 and 500 lx varies *I*_B_ from 21 up to 52 μA and changes *f*_0_ of the generator between 70 and 154 kHz. The difference between theory and measurement results reaches only ±5% in most of the observed range.

## Figures and Tables

**Table 1:** Comparison of recent concepts of electronically linearly tunable generators (features of real experiments documented for all possible cases, if available).

Reference	Number of Passive Elements (in Lab. Experiments)	Number of Active Elements	Number of IC Packages (in Laboratory Experiments)	Compact IC Device Including All Active Elements	Maximal Current (*I*_Cmax_) Charging *C* Independent on Linear Operation of the AE	Integrator Uses Different Part than OTA (Where *I*_Cmax_ ≅ *I*_bias_)	Type of Active Element(s)	Number of Internal Functional Subparts of Active Device	Classification of Active Element(s) Used in Verification	Verification	Power Consumption
[5]	3	3	3	No	Yes	No	OTA	N/A	standard	M	N/A
[6]	3	3	N/A	No	Yes	No	OTA	N/A	standard	S	N/A
[7]	1	2	N/A	No	Yes	No	MO-CTTA	2	hypothetical	S	N/A
[8]	1	2(8)	N/A	No	Yes	No	MO-CCCDTA	2	hypothetical	S	N/A
[9]	1	2	N/A	No	Yes	No	MO-CCCCTA	2	hypothetical	S	1.6 mW
[10]	8	2	1	Yes	Yes	Yes	UCC + CCII	N/A	fabricated (CMOS)	S	N/A
[11]	3	2	N/A	No	Yes	No	VDBA	2	hypothetical	S	N/A
[12]	3	1	5	No	Yes	No	ZC-CG-VDCC	2	hypothetical	S	6.3 mW
[13]	1	1	8	No	Yes	No	ZC-CG-VDCC	2	hypothetical	M	N/A
[14]	4	3	3	No	No	Yes	ECCII + VGA + OPAMP	N/A	standard	S	N/A
[15]	2	1	N/A	No	No	Yes	CG-CDVA	2	hypothetical	S	N/A
[16]	2	3	3	No	Yes	Yes	DT + VGA	N/A	standard	M	N/A
[17]	2	2	2	No	No	Yes	ECCII + VGA	N/A	standard	M	N/A
[18]	2(4)	1	7	No	Yes	No	MO-DXCCTA	2	hypothetical	M	N/A
[19]	2(4)	1	6	No	Yes	No	MO-DVCCTA	2	hypothetical	M	226 mW
[20]	3	2	N/A	No	Yes	No	MO-VDTA	2	hypothetical	S	14.3 mW
[21]	2(4)	1	8	No	Yes	No	MO-DXCCTA	2	hypothetical	S	1 mW
[22]	1(2)	1	5	No	Yes	No	MO-CIDITA	2	hypothetical	M	^1^
[23]	1(2)	1	4	No	Yes	No	MO-CFDITA	2	hypothetical	M	^2^
* [24]	2(3)	4(5)	2(3)	No	No	Yes	CFOA	N/A	standard	M	458 mW
[25]	4	2	2	No	No	Yes	CCII/CCCII + OTA	N/A	standard	M	192 mW
* [26]	4	2(3)	6(9)	No	No	Yes	DVCC	1	hypothetical	M	763 mW
Proposed											
Figure 1a	3	2	1	Yes	Yes	Yes	CCCII + OTA	N/A	fabricated (CMOS I3T)	M	67 mW
Figure 1b	3	3	1	Yes	Yes	Yes	CCCII + OTA + VF	N/A	fabricated (CMOS I3T)	M	86 mW

^1^ Result 0.5 mW available for simulations only (not for experiment), ^2^ result 1.45 mW available for simulations only (not for experiment); * these solutions are only partially electronically controlled (duty cycle tested); S: Simulated; M: Measured; N/A: Not available; Note: Information about the area of active elements in cited works is not available. CCCII: Current controlled current conveyor; CCII: Current conveyor of second generation; CFOA: Current feedback operational amplifier; CG-CDVA: Controlled gain current and differential voltage amplifier; DT: Diamond transistor; DVCC: Differential voltage current conveyor; ECCII: Electronically controllable CCII; MO-CCCCTA: Multiple-output current controlled current conveyor transconductance amplifier; MO-CCCDTA: Multiple-output current controlled current differencing transconductance amplifier; MO-CFDITA: Multiple-output current follower differential input transconductance amplifier; MO-CTTA: Multi-output current through transconductance amplifier; MO-CIDITA: Multiple-output current inverting differential input transconductance amplifier; MO-DVCCTA: Multiple-output differential voltage current conveyor transconductance amplifier; MO-DXCCTA: Dual-X current conveyor transconductance amplifier; OPAMP: Operational amplifier; UCC: Universal current conveyor; VF: Voltage follower; VDBA: Voltage differencing buffered amplifier; VGA: Variable gain amplifier; ZC-CG-VDCC: Z-copy controlled gain voltage differencing current conveyor.
